# Dietary Inflammatory Index, Dietary Non-Enzymatic Antioxidant Capacity, and Colorectal and Breast Cancer Risk (MCC-Spain Study)

**DOI:** 10.3390/nu11061406

**Published:** 2019-06-21

**Authors:** Mireia Obón-Santacana, Dora Romaguera, Esther Gracia-Lavedan, Amaia Molinuevo, Esther Molina-Montes, Nitin Shivappa, James R. Hebert, Adonina Tardón, Gemma Castaño-Vinyals, Ferran Moratalla, Elisabet Guinó, Rafael Marcos-Gragera, Mikel Azpiri, Leire Gil, Rocío Olmedo-Requena, Macarena Lozano-Lorca, Juan Alguacil, Tania Fernández-Villa, Vicente Martín, Antonio J Molina, María Ederra, Conchi Moreno-Iribas, Beatriz Perez, Nuria Aragonés, Adela Castello, José Mª Huerta, Trinidad Dierssen-Sotos, Inés Gómez-Acebo, Ana Molina-Barceló, Marina Pollán, Manolis Kogevinas, Victor Moreno, Pilar Amiano

**Affiliations:** 1 Oncology Data Analytics Program (ODAP), Catalan Institute of Oncology (ICO), L’Hospitalet del Llobregat, 08908 Barcelona, Spain; mobon@idibell.cat (M.O.-S.), fmoratalla@iconcologia.net (F.M.), e.guino@iconcologia.net (E.G.); v.moreno@iconcologia.net (V.M.); 2 ONCOBELL Program, Bellvitge Biomedical Research Institute (IDIBELL), L’Hospitalet de Llobregat, 08908 Barcelona, Spain; 3 Consortium for Biomedical Research in Epidemiology and Public Health (CIBERESP), 28029 Madrid, Spain; esther.gracia@isglobal.org (E.G.-L.); au-molinuevo@euskadi.eus (A.M.); atardon@uniovi.es (A.T.); gemma.castano@isglobal.org (G.C.-V.); rmarcos@iconcologia.net (R.M.-G.); rocioolmedo@ugr.es (R.O.-R.); juan.alguacil@dbasp.uhu.es (J.A.); vicente.martin@unileon.es (V.M.); maria.ederra.sanz@cfnavarra.es (M.E.); bperez@isciii.es (B.P.); nuria.aragones@salud.madrid.org (N.A.); acastello@isciii.es (A.C.); jmhuerta.carm@gmail.com (J.M.H.); trinidad.dierssen@unican.es (T.D.-S.); ines.gomez@unican.es (I.G.-A.); mpollan@isciii.es (M.P.); manolis.kogevinas@isglobal.org (M.K.); epicss-san@euskadi.eus (P.A.); 4 Instituto de Salud Global de Barcelona (ISGlobal), 08036 Barcelona, Spain; 5 Instituto de Investigación Sanitaria Illes Balears (IdISBa), 07120 Palma de Mallorca, Spain; 6 CIBER Fisiopatología de la Obesidad y Nutrición (CIBEROBN), 28029 Madrid, Spain; 7 Departament de Ciències Experimentals i de la Salut, Universitat Pompeu Fabra (UPF), 08002 Barcelona, Spain; 8 Genetic and Molecular Epidemiology Group, Spanish National Cancer Research Center (CNIO), 28029 Madrid, Spain; memolina@cnio.es; 9 Centro de Investigación Biomédica en Red de Cáncer (CIBERONC), 28029 Madrid, Spain; 10 Cancer Prevention and Control Program, University of South Carolina, Columbia, SC 29208, USA.; shivappa@email.sc.edu (N.S.); JHEBERT@mailbox.sc.edu (J.R.H.); 11 Department of Epidemiology and Biostatistics, Arnold School of Public Health, University of South Carolina, Columbia, SC 29208, USA; 12 Department of Nutrition, Connecting Health Innovations LLC (CHI), Columbia, SC 29201, USA; 13 Department of Medicine, University of Oviedo, 33006 Oviedo, Spain; 14 IMIM (Hospital del Mar Medical Research Institute), 08003 Barcelona, Spain; 15 Department of Clinical Sciences, Faculty of Medicine, University of Barcelona, 08007 Barcelona, Spain; 16 Genetics and Cancer Prevention Group, Epidemiology Unit and Girona Cancer Registry, Descriptive Epidemiology, IdIbGi, Catalan Institute of Oncology, 17007 Girona, Spain; 17 Public Health Division of Gipuzkoa, 20003 San Sebastian, Spain; koor-tolosa@euskadi.eus (M.A.); l-gil@euskadi.eus (L.G.); 18 Biodonostia Research Institute, 20014 San Sebastian, Spain; 19 Department of Preventive Medicine and Public Health, School of Medicine, University of Granada, 18016 Granada, Spain; macarenalozano@ugr.es; 20 Instituto de Investigación Biosanitaria de Granada ibs.GRANADA, 18014 Granada, Spain; 21 Centro de Investigación en Recursos Naturales, Salud y Medio Ambiente (RENSMA), Universidad de Huelva, 21071Huelva, Spain; 22 The Research Group in Gene - Environment and Health Interactions (GIIGAS) / Institut of Biomedicine (IBIOMED), Universidad de León, 24071 León, Spain; tferv@unileon.es (T.F-V.); ajmolt@unileon.es (A J.M.); 23 Faculty of Health Sciences, Department of Biomedical Sciences, Area of Preventive Medicine and Public Health, Universidad de León, 24071 León, Spain; 24 Navarra Public Health Institute, 31003 Pamplona, Spain; mc.moreno.iribas@cfnavarra.es; 25 Navarra Institute for Health Research (IdiSNA), 31003 Pamplona, Spain; 26 REDISSEC Red de Investigación en Servicios de Salud en Enfermedades Crónicas, 28029 Madrid, Spain; 27 Cancer & Environmental Epidemiology Unit, Department of Epidemiology of Chronic Diseases, National Centre for Epidemiology, Carlos III Institute of Health, 28029 Madrid, Spain; 28 Department of Health of Madrid, Cancer Epidemiology Section, Public Health Division, 28029 Madrid, Spain; 29 Department of Surgery and Medical and Social Sciences. Alcalá de Henares University, 28801 Alcalá de Henares, Madrid, Spain; 30 Department of Epidemiology, Murcia Regional Health Council, IMIB-Arrixaca, 30008 Murcia, Spain; 31 Preventive Medicine, Universidad de Cantabria—IDIVAL, 39011 Santander, Spain; 32 Cancer and Public Health Area, FISABIO—Public Health; 46020 Valencia, Spain; molina_anabar@gva.es

**Keywords:** colorectal cancer, breast cancer, diet, dietary inflammatory index, antioxidants, NEAC, case-control study, MCC-Spain

## Abstract

Inflammation and antioxidant capacity have been associated with colorectal and breast cancer. We computed the dietary inflammatory index (DII^®^), and the total dietary non-enzymatic antioxidant capacity (NEAC) and associated them with colorectal and breast cancer risk in the population-based multi case-control study in Spain (MCC-Spain). We included 1852 colorectal cancer and 1567 breast cancer cases, and 3447 and 1486 population controls, respectively. DII score and NEAC were derived using data from a semi-quantitative validated food frequency questionnaire. Unconditional logistic regression models were used to estimate odds ratios (OR) and 95% confidence intervals (95%CI) for energy-adjusted DII (E-DII), and a score combining E-DII and NEAC. E-DII was associated with colorectal cancer risk (OR = 1.93, highest quartile versus lowest, 95%CI:1.60–2.32; *p*-trend: <0.001); this increase was observed for both colon and rectal cancer. Less pronounced increased risks were observed for breast cancer (OR = 1.22, highest quartile versus lowest, 95%CI:0.99–1.52, *p*-trend: >0.10). The combined score of high E-DII scores and low antioxidant values were associated with colorectal cancer risk (OR = 1.48, highest quartile versus lowest, 95%CI: 1.26–1.74; *p*-trend: <0.001), but not breast cancer. This study provides evidence that a pro-inflammatory diet is associated with increased colorectal cancer risk while findings for breast cancer were less consistent.

## 1. Introduction

Colorectal cancer (CRC) is the third most common cancer worldwide [1]. Beyond non-modifiable risk factors such as age, male sex, family history of CRC, and genetic predisposition [2], epidemiological studies have identified a number of modifiable factors that have a direct impact on CRC risk, for example cigarette smoking, which increases risk [3], and regular use of aspirin, which decreases risk [4]. Regarding nutritional factors, consuming processed and red meat, alcoholic beverages, and being overweight or obese increases the risk of developing CRC; whereas being physically active, consuming whole grains, foods rich in fiber including fruits and vegetables, dairy products, and calcium supplements decrease CRC risk [5]. 

Among females, breast cancer (BC) and CRC are the two most frequently diagnosed cancers [6,7]. Age, BRCA1 or BRCA2 genes mutations, family history of BC or ovarian cancer, radiation, hormonal factors, physical inactivity, alcohol consumption, tobacco smoking and physical inactivity are important risk factors for BC [8,9]. The role of diet, however, remains controversial [9].

Despite that CRC and BC are different cancers affecting different organs, they share similar risk factors. Scientific evidence has shown that chronic inflammation and oxidative stress predispose the pathogenesis of numerous diseases, including CRC and BC [10,11,12,13]. Additionally, it has been observed that oxidative stress produces DNA damage and increases cancer risk, partially mediated through inflammation, suggesting that both mechanisms are also related [14,15]. 

Diet may play a role in the regulation of chronic inflammation, as shown by the relation between dietary factors and blood levels of inflammatory markers [16,17,18]. There have been several approaches to assess the inflammatory potential of the overall diet, beyond the study of single nutrients and foods, and the most widely used is the dietary inflammatory index (DII^®^), which was specifically designed to assess and offer quantitative information about the inflammatory potential of the diet [19]. The DII has been shown to be associated with various biomarkers of inflammation in several studies [20,21,22] including those in Europe [23,24,25]. The relation between diet and oxidative stress also has been evaluated, but the molecular mechanisms are still under study [26]. The non-enzymatic antioxidant capacity (NEAC) has been proven to be a useful tool to estimate the total dietary antioxidant content, as it takes into account all the antioxidants and bioactive compounds present in the diet and the synergistic effects between them [27,28]. 

Numerous epidemiological studies have assessed the association between DII and energy-adjusted (E-DII) scores and CRC and BC risk, supporting the hypothesis that a pro-inflammatory diet is related to CRC risk [29]. The evidence for BC is less clear [30,31,32]. The role of NEAC has also been evaluated in several cancer sites, including CRC and BC. Despite that some studies indicated no clear association between NEAC and CRC risk [33,34], others have observed statistically significant decreased risk for CRC [28,35], as well as for BC risk [36,37]. 

Thus far, studies essentially have focused on assessing the role of the inflammatory potential of the diet on cancer risk, without considering the plausible combined effect of a global indicator of NEAC. Using the newly constructed E-DII in the population-based multi-case-control (MCC)-Spain study our aim is to analyze the association between the inflammatory potential of the diet, and the risk of developing BC and CRC, as well as among tumor subtypes. A second aim of the present study is to elucidate the relation between E-DII and NEAC, and to evaluate whether having an anti-inflammatory and antioxidant diet affects the risk of developing CRC and BC.

## 2. Materials and Methods 

### 2.1. Study Population and Design

MCC-Spain is a population-based multicenter case-control study designed to investigate the etiology of the principal cancer sites (colorectal, breast, prostate, and gastric tumors, and chronic lymphocytic leukemia) in adults. The study design and protocol have been described in detail elsewhere [38]. The MCC-Spain enrolled cases and controls from 12 Spanish provinces (Asturias, Barcelona, Cantabria, Girona, Granada, Guipúzcoa, Huelva, León, Madrid, Murcia, Navarra, and Valencia) from 2008 to 2013. All participants who were eligible to take part in the study and agreed to participate signed an informed consent. The study protocol of the MCC-Spain was approved by all the Ethics Committees of the participating institutions and followed national and international directives on ethics and data protection [38]. 

**Figure 1:**
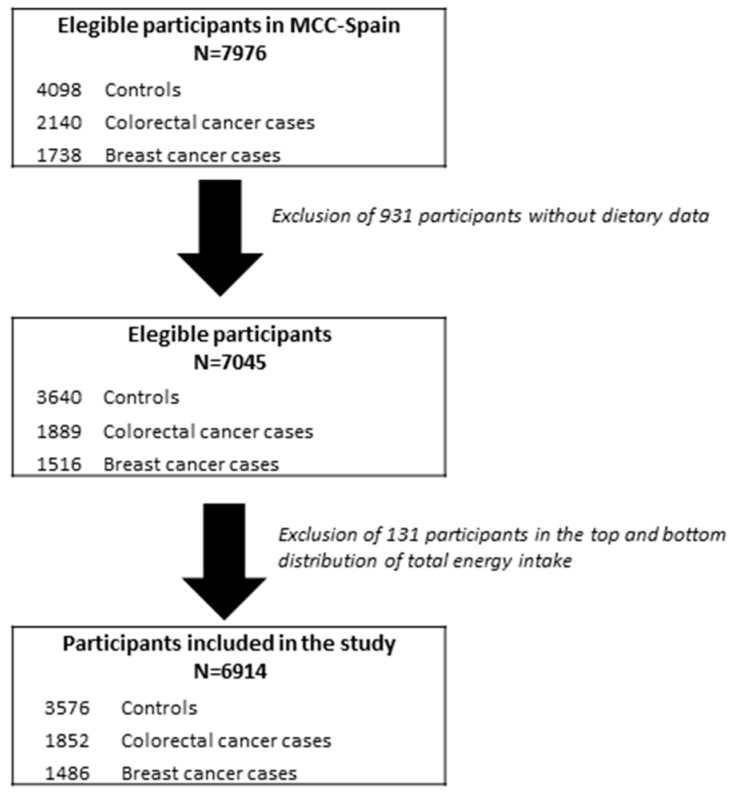
Flow chart of study population selection.

The CRC sub-study included 1852 cases and 3347 controls, and the BC sub-study included 1486 cases and 1652 controls (Figure 1). Newly diagnosed cancer cases were ascertained through the oncologic or digestive units of the participating hospitals. All CRC and BC cases were histologically confirmed. CRC cases were classified according to the International Classification of Diseases 10th Revision as C18, C19, C20, D01.0, D01.1, and D01.2 (including cancer of the colon or rectum) whereas BC cases were classified as C50, D05.1, and D05.7. Controls were randomly selected from lists of primary care centers located in the catchment area of hospitals from where the cases came. They were frequency matched to cases on age, sex, and region. As reported elsewhere, the mean response rate of controls was 53%, and differed by region [38].

### 2.2. Epidemiological Data Collection

Face-to-face interviews were carried out at baseline by trained personnel using a structured computerized epidemiological questionnaire to assess data on socio-demographic, lifestyle, environmental exposure, residential history, personal/family medical history, drug use, and weight information at different ages. The questionnaire is available at http://www.mccspain.org. 

Information on dietary data was assessed using a self-administered semi-quantitative food frequency questionnaire (FFQ), which was an adapted version of a Spanish-validated FFQ [39] as it was modified to include regional products. Details on the validation study of this FFQ are provided elsewhere. In brief, the de-attenuated correlation coefficients between the second questionnaire and diet records ranged from 0.45 for vitamin A to 0.91 for alcohol [40]. The MCC-study FFQ included portion sizes and photos to assess dietary information with the timeframe referring to the preceding year. Further, cross-check questions on food groups intakes were designed to adjust the frequency of food consumption and to reduce misreporting of food groups with large numbers of items. A total of 140 items were obtained. Information on total energy intake and intake of both macronutrients and micronutrients, as well as alcohol consumption were derived from Spanish food composition tables and other specific sources [41]. 

### 2.3. Dietary Non-Enzymatic Antioxidant Capacity and Dietary Inflammatory Index Assessment

The assessment of the dietary NEAC in the MCC-Study has been previously described in detail [28]. Briefly, for the present study, dietary NEAC was estimated using the Trolox equivalent antioxidant capacity (TEAC-ABTS, referred from now on as TEAC; mmol TE/Kg) using published values of NEAC content in food, and was assessed with and without coffee information. The MCC-study FFQ food items were matched with the corresponding dietary NEAC values, and the average daily NEAC consumption (using TEAC) was estimated for each participant. 

DII scores were calculated using a method previously reported by Shivappa et al. [19]. Briefly, the scoring algorithm based on extensive review of the literature focused on the effect of diet on six inflammatory biomarkers (IL-1β, IL-4, IL-6, IL-10, TNF-α, and CRP) from 1950 to 2010. A total of 45 food parameters, including macronutrients and micronutrients, were scored according to whether they increased (+1), decreased (-1), or had no effect (0) on these inflammatory biomarkers. These scores were weighted based on study design. To avoid the arbitrariness resulting from simply using raw consumption amounts, intakes of foods and nutrition were standardized to a representative range of dietary intake based on actual human consumption in 11 populations living in different countries across the world that provided an estimate of a mean and standard deviation for each parameter. These values were converted to a proportion (with values from 0 to 1). Each proportion was doubled, and then 1 was subtracted to achieve a symmetrical distribution around a mean of ≈0. Each of these values was then multiplied by an overall food parameter-specific inflammation score. All the food parameter-specific DII scores were summed to create the overall DII scores for each subject. Energy-adjusted DII (E-DII) scores were calculated by converting raw dietary components to amount per 1000 kcal. To compute the E-DII scores, we relied on an energy-adjusted global database. Higher E-DII scores indicate more pro-inflammatory diets, while lower E-DII scores represent anti-inflammatory diets. In this study, data were available for a total of 30 food parameters (carbohydrate, protein, total fat, alcohol, fiber, cholesterol, saturated fatty acid, monounsaturated fatty acid, polyunsaturated fatty acid, vitamin A, vitamin B1, vitamin B2, vitamin B3, vitamin B6, vitamin B12, vitamin C, vitamin D, vitamin E, folic acid, iron, magnesium, zinc, anthocyanidins, flavan3ols, flavones, flavonols, flavonones, isoflavones, garlic, and onion) to calculate the E-DII scores. E-DII scores were used in all analyses in this study.

### 2.4. Statistical Analyses

Baseline dietary and sociodemographic characteristics were examined using means and standard deviations (SD) for continuous variables and percentages for categorical variables by CRC and BC cases and controls, and across E-DII quartiles. Differences between cases and controls were assessed using Student’s *t*-test for continuous variables normally distributed, Wilcoxon rank-sum test for continuous variables non-normally distributed, and Pearson χ² test for categorical variables. The E-DII was analyzed as a continuous variable (one-unit increment) and as a categorical variable, expressed as quartiles based on the sex-specific distribution in the control group (CRC or BC controls). The first E-DII quartile was treated as reference category (meaning lowest inflammatory potential of the diet). 

The odds ratios (OR) and the corresponding 95% confidence intervals (95%CI) for the association between E-DII and CRC and BC risk were estimated using unconditional logistic regression models. Analyses also were conducted separately by CRC anatomic subsites (colon and rectum) and BC subtypes (HR+, HER2+, TN). Tests for dose-response trend were estimated by including the exposure variable as continuous ordinal (scored from 1 to 4) in the regression model. 

Effect-measure modification by NSAIDs (yes, no), physical activity (inactive, active), BMI (<25, ≥25 kg/m^2^), and smoking status (never, ever) was evaluated by including interaction terms and by stratified analysis. The models with and without the interaction terms were compared using the likelihood ratio test (LRT). 

For analyses where CRC was the main outcome, two different models are presented: (1) A simple model adjusting for age, sex, study area, and educational level (less than primary, primary, high school, university); (2) a final model, additionally adjusting for family history as first-degree relative of CRC (no, yes, unknown), smoking status (never, current, former), BMI (kg/m^2^) one year before recruitment, leisure-time physical activity calculated for the last 10 years of life, excluding the last 2 years previous to the interview (inactive, moderately active, active, very active), and non-steroidal anti-inflammatory drugs use (NSAIDs; yes, no, unknown).

Likewise, two different models were evaluated to study the relation between E-DII and BC risk: (1) A simple model (previously defined, excepting sex); (2) a final model further adjusted by family history first degree of BC (no, yes, unknown), smoking status, BMI, physical activity, hormonal replacement therapy use (HRT; no, yes, unknown), oral contraceptive use (OC; no, yes, unknown), age at menarche (<13, ≥13 years, unknown), age at first pregnancy (no children, <20, 20–24, 25–29, >29 years), number of children, and menopausal status (premenopausal, postmenopausal).

Potential effect-measure modification of the association between E-DII and BC risk also was assessed using a LRT in the mutually adjusted model by physical activity, BMI, smoking status, menopausal status (premenopausal, postmenopausal), HRT use (yes, no), and OC use (yes, no).

Three sensitivity analyses for both CRC and BC final models were performed: 1) Excluding those participants who had more than 6 months between the data of diagnosis and the interview (final models included 1596 CRC cases and 1140 BC cases); 2) further adjusting the final CRC and BC models by energy intake from non-alcohol sources and alcohol consumption; 3) restricting the analyses to non-drinkers. 

To assess the second aim of the present study, two approaches were conducted. First, an inflammatory and antioxidant profile was designed, and it was used as the main exposure variable in both CRC and BC final models. A value of 0, 1, 2, and 3 was assigned to the first, second, third, and fourth quartile of E-DII (lower scores indicate lower inflammatory potential of diet), whereas a value of 3, 2, 1, and 0 was assigned to the first, second, third, and fourth quartile of NEAC (using TEAC; higher score indicates higher antioxidant capacity of diet). When CRC was evaluated, quartiles values were sex-specific. For each participant, the values received were summed to assess the profile score. The profile ranged from 0 (less inflammatory and high antioxidant) to 6 (high inflammatory and less antioxidant). Linear trend tests were used to calculate the OR for the profile as a continuous variable (1 point-increment). Second, a combined categorical variable including both indicators (E-DII and TEAC) was created using median values (sex-specific for CRC). This four-category variable was used as the main exposure variable in final models. Those participants classified having high antioxidant and low inflammatory potential of diet were selected as the reference category. Potential effect-measure modification of the association between E-DII+NEAC score and both cancer outcomes was evaluated by vegetable intake (low, high), all meat intake (low, high), and fiber intake (low, high).

Statistical analyses were conducted using R: A language and environment for statistical computing, version 3.5 (R Core Team, 2018). All statistical tests were two-sided and statistical significance was set at *p* < 0.05.

## 3. Results

### 3.1. Characteristics of Cases and Controls in the MCC-Study

The present study included 1852 CRC and 1486 BC cases, and 3447 and 1652 population controls, respectively. The main characteristics of cases and controls are presented in Table 1. Briefly, CRC cases compared to controls had higher E-DII scores, indicating a more pro-inflammatory diet, tended to be older, heavier, and less active, and more frequently classified as having a low education and a family history of CRC (*p* < 0.001). BC cases compared to controls also had higher E-DII scores, tended to be younger, and reported more frequently being smokers, premenopausal, and having a first-degree history of BC (*p* < 0.001). Similar distributions were observed among BC cases and controls regarding HRT, OC use, and age at first pregnancy (*p* > 0.05). 

Characteristics of participants in the control group across quartiles of E-DII are shown in Table 2. Participants in the highest quartile (Q4) were younger, consumed more calories (without considering alcohol) and alcohol, reported being current smokers, and less active compared with those in the lowest quartile (Q1) (*p* < 0.001). Among female controls, those classified in the Q4 compared with Q1 tended to be premenopausal and were OC but not HRT users. No differences were observed across quartiles regarding age at menarche, age at first pregnancy, and number of children (all *p* > 0.30).

### 3.2. E-DII and Colorectal Cancer Risk

The OR and 95% CI for the association between E-DII and CRC risk, as well as according to the location of the tumor and the stratified results, are presented in Table 3. A statistically significant increased CRC risk was observed with increasing E-DII score per 1-point increment both in the minimally (OR_E-DII_: 1.15, 95%CI: 1.11–1.19) and fully adjusted models (OR_E-DII_: 1.14, 95%CI: 1.10–1.18). Participants classified at the fourth E-DII quartile were at higher risk of developing CRC (OR_Q4vsQ1_: 1.93, 95%CI: 1.60–2.32; *p*-value for trend: <0.001). This association was consistent when colon and rectal CRC subtypes were evaluated using the continuous E-DII variable per 1-point increment (OR_E-DII_: 1.13, 95%CI: 1.08–1.17, and OR_E-DII_: 1.17, 95%CI: 1.11–1.22; respectively). Results remained unchanged when we performed the three sensitivity analyses: (1) Excluding those participants who had more than six months between the data of diagnosis and the interview (Table 3); (2) adjusting the final model by energy intake from non-alcohol sources and alcohol consumption (data not shown); and (3) restricting the analyses to non-drinkers (data not shown).

The E-DII score was statistically significant positively associated with CRC risk in both males and females (*p*-value for trend: <0.001; LRT *p*-value: 0.04), but higher ORs were observed in men (OR_Q4vsQ1_: 2.14, 95%CI: 1.68–2.73) than in women (OR_Q4vsQ1_: 1.57, 95%CI: 1.16–2.13) (Table 3).

Effect-measure modification by BMI, physical activity, NSAIDs/aspirin use, and tobacco smoking was evaluated as these factors are related to inflammation and to CRC risk (Table 3). None of the LRT *p*-values showed evidence for heterogeneity (all LRT *p*-values > 0.25).

### 3.3. E-DII and Breast Cancer Risk

Table 4 displays the crude and adjusted OR and 95% CI for the association between E-DII and overall BC risk and BC subtypes, in addition to stratified analyses. A statistically significant increased BC risk was observed in the minimally adjusted model (OR_E-DII_: 1.05, 95%CI: 1.01–1.09; OR_Q4vsQ1_: 1.25, 95%CI: 1.02–1.55; *p*-value for trend: 0.06) but not in the fully adjusted model, where confidence limits overlapped unity (OR_E-DII_: 1.04, 95%CI: 1.00–1.08; OR_Q4vsQ1_: 1.22, 95%CI: 0.99–1.52; *p*-value for trend: 0.10). Sensitivity analyses did not alter the final model results and even when we excluded those BC cases that had more than six months between the data of diagnosis and the interview, results were not statistically significant (Table 4). 

BC subtypes (HR+, HER2+, TN) also were investigated. No associations were found between E-DII (measured both as a continuous and categorical) and HR+ and TN subtypes. A statistically significant increased HER2+ risk was observed when E-DII was evaluated as a categorical variable (OR_Q4vsQ1_: 1.56, 95%CI: 1.01–2.04); however, there was no evidence of a linear dose response trend (*p*-value: 0.24) (Table 4).

Despite observing some individual statistically significant ORs among inactive (OR_Q4vsQ1_: 1.48, 95%CI: 1.04–2.10, *p*-value for trend: >0.13) and postmenopausal women (OR_E-DII_: 1.06, 95%CI: 1.01–1.12), no consistent evidence for effect-measure modification was observed by menopausal status, HRT and OC use, BMI, physical activity, and tobacco smoking (all LRT *p*-values > 0.15) (Table 4).

### 3.4. E-DII, NEAC, and Colorectal and Breast Cancer Risk

The correlation coefficient between E-DII and NEAC in the MCC-Spain study was −0.32 (*p*-value < 0.001). The ORs and 95% CI for the association between the inflammatory and antioxidant profile (combining information on E-DII and dietary NEAC) and CRC and BC risk are presented in Table 5. The inflammatory and antioxidant profile ranged from 0 (less inflammatory, high antioxidant) to 6 (high inflammatory and less antioxidant). The continuous variable (1-point increment in the E-DII+NEAC profile) included 1798 CRC cases and 3312 controls and showed a statistically significant 10% increasing risk. Participants classified at the highest category of the score (including 499 cases and 790 controls) compared to the first category (including 642 cases and 1404 controls) had higher risk of developing CRC (OR_Q4vsQ1_: 1.48, 95%CI: 1.26–1.74; *p*-value for trend: <0.001). 

Results regarding BC risk showed a non-statistically significant increased risk when the profile was evaluated continuously (OR: 1.02, 95%CI: 0.98–1.07), as well as for participants classified at the fourth quartile (399 cases and 388 controls) compared to the first quartile (563 cases and 673 controls) (OR_Q4vsQ1_: 1.09, 95%CI: 0.90–1.32, *p*-value for trend: 0.39). Similar results for both outcomes were obtained when the combined categorical variable was used as the main exposure variable (Supplemental Table S1).

No evidence for effect-measure modification was observed in CRC models by low vs. high fiber, vegetables, and meat consumers (all LRT *p-*values > 0.05; Supplemental Table S2). Likewise, in BC models, none of the LRT *p*-values showed evidence for heterogeneity when low vs. high fiber and vegetables consumers were evaluated (LRT *p*-values > 0.28; Supplemental Table S2). However, there was statistically significant heterogeneity by meat intake (LRT *p-*value: 0.004). Those women who had higher values of meat consumption (above the median: ≥67 grams/day), had a 9% increased BC risk for each increment in the E-DII+NEAC score profile. High meat consumers classified at the highest category of the score (including 270 cases and 207 controls) had higher risk of developing BC (OR_Q4vsQ1_: 1.43, 95%CI: 1.10–1.85; *p*-value for trend: <0.001). 

## 4. Discussion

This large case-control study in Spain provided further evidence that a pro-inflammatory diet (measured by the widely used E-DII) was clearly associated with increased CRC risk, and this risk was persistently shared across different tumor locations and all subgroups that were evaluated. Results regarding risk of BC do not support the hypothesis of an association; although non-statistically significant increased risks were observed when E-DII was analyzed both as a continuous variable and by quartiles. This study, including both outcomes, observed no changes when sensitivity analyses were carried out and did not observe evidence for effect-measure modification when subgroups were analyzed. 

The epidemiologic evidence on the association between DII and CRC risk has been reported in 4 prospective [42,43,44,45], 5 case-controls studies [46,47,48,49,50], and several meta-analyses [29,30,51,52]. Despite the type of study design (case-control or prospective cohorts), the differences in the total number of food/nutrients items included in the previously reported DII scores, and the different DII-approaches used as the main exposure variable (with and without energy-adjustment), results reflect that a greater dietary inflammatory potential is related to CRC risk, and this effect tends to be more pronounced in men than in women. The present study is the first epidemiologic study that replicates these results using the E-DII score in Spanish populations.

Several dietary components and dietary patterns have been associated with CRC risk [5]. A total of 9 of the 45 possible items that comprise the DII score are pro-inflammatory: energy, carbohydrates, protein, total fat, saturated fat, trans fat, cholesterol, vitamin B_12_, and iron [19]. Most of these items are related to Western-style diets, which have been associated with inflammation [53]. In agreement with our results, a recent paper from our colleagues, Castelló, et al showed that a high adherence to the Western dietary pattern was statistically significant positive associated with overall CRC risk (OR_Q4vsQ1_: 1.50, 95%CI: 1.20–1.87), whereas a high adherence to the Mediterranean diet was inversely associated (OR_Q4vsQ1_: 0.65, 95%CI: 0.53–0.80). Likewise, similar relative risks were observed for both patterns among males and females [54]. 

The development and progression of BC has been linked, among other parameters, to inflammation [55]. The evidence regarding the association between DII and overall BC risk is still inconclusive despite being evaluated in several prospective cohorts [31,32,56,57,58], case-control studies [59,60,61,62,63], and cancer-specific and general meta-analyses [30,64,65,66]. Although our estimates suggested increased relative risks for the association between E-DII and overall BC, the 95% CIs included the null value. Our highest quartile of E-DII did not include very high E-DII values, and this may have limited our capability to observe statistically significant associations. Moreover, two recent reviews and meta-analysis have observed significant associations between high vs. low inflammatory diet in postmenopausal women but no for premenopausal women [65,66]. The present study also observed a significant association among postmenopausal women when E-DII was evaluated continuously, but no effect-measure modification was observed by menopausal status. Likewise, the observed results may have been influenced by alcohol consumption. During the calculation of the DII and E-DII score, alcohol is negatively weighted (meaning low inflammation), and alcohol consumption is considered an important risk factor for BC [67]. We performed a sensitivity analysis further adjusting for alcohol intake and total energy (without alcohol), and we restricted the analysis to non-drinkers; however, results were not altered. Further studies evaluating BC risk or survival should consider calculating the DII or the E-DII without accounting for alcohol intake. 

Our study adds value to the scientific evidence because, up to now, none of the epidemiological studies cited above have evaluated the association between E-DII and cancer risk taking into account the total anti-oxidant dietary content. Dietary NEAC intake has been associated with a decreased risk of CRC in two large case-control studies, including the MCC-study [28,35]; whereas inconclusive results were observed in two prospective cohorts [33,34]. The association between dietary NEAC and BC is also doubtful. To our knowledge, only two studies have reported results: A prospective study showing a significant decreased risk [37] and a case-control study, which observed null associations [36]. Inflammation and oxidative stress are two mechanisms that have been independently related to cancer risk [68,69]; however, they are closely tied [14,70]. The profile score, including information on E-DII and dietary NEAC, showed consistent and significant increased effect estimates of CRC in all categories, as well as per 1-point increment in the score, suggesting that both E-DII and NEAC are linked to CRC risk. However, relative risks were slightly attenuated compared to those obtained when E-DII was used as the main exposure variable. Results for BC risk remained elevated, but not statistically significant. Likewise, a similar pattern was observed; i.e., relative risks were somewhat lower. Similar relative risks were observed when a combined variable (low E-DII and high TEAC vs high E-DII and low TEAC) was used instead of the profile score. This may indicate that E-DII plays a stronger effect on CRC and BC risk than NEAC, or that the E-DII and NEAC profile needs to be assessed in other ways. When the analyses were restricted to high/low consumers of vegetables, fiber, and meat, no evidence for heterogeneity was observed for CRC risk. Similar results were observed for BC risk; however, we found a statistically significant interaction between E-DII+NEAC score and meat intake. Women in the highest quartile of the score and were higher meat consumers were at higher BC risk . Although meat intake *per se* is not taken into account in the construction of the DII, meat-derived macronutrients and micronutrients are included as pro-inflammatory factors. Thus, higher consumption of meat results in higher E-DII values (and consequently higher E-DII+NEAC values). In addition, this category included more BC cases than controls (270 vs. 207). Meat intake has also been associated with increased BC risk in the MCC-Spain study, especially among postmenopausal women [71]. These findings could indicate a synergistic effect of meat consumption and having a pro-inflammatory and pro-oxidant diet on BC risk. Nonetheless, further research is warranted in this field to confirm these findings. 

This study is based on a relatively large sample size that enabled us to investigate specific colorectal and breast tumor subtypes, as well as different subgroups (i.e., menopausal status, physical activity, NSAIDs/aspirin use). Additionally, we have used a valid tool to assess the inflammatory potential of the diet, which has been investigated in many different studies, regions and different outcomes [72].

Notwithstanding its strengths, there are some weaknesses in our study that should be mentioned. Case-control designs are prone to selection and recall biases. The MCC-study was designed with the goal of minimizing selection biases by recruiting population-based controls and cases [38]. Regarding recall bias, the dietary information collected at recruitment referred to the preceding year, and sensitivity analysis were carried out excluding CRC and BC cases diagnosed within less than six months between the date of diagnosis. When we did this, results did not differ substantially. Measurement error also may be present in our dietary data; nonetheless, the MCC-study assessed the dietary information by using a self-reported FFQ validated for Spanish population. As in that work, the DII derived from the MCC-Study was energy-adjusted and included only 30 of the 45 food/nutrient parameters, and we expressed the E-DII as quartiles based on the sex-specific distribution within the control group. Thus, direct comparison to other E-DII/DII quantile categorizations is challenging: Most of the studies assessed the inflammatory potential of the diet using the DII (energy was included in the score or as a covariate in final models); depending on the study, the E-DII/DII score was estimated including different number of food/nutrient parameters; other approaches have been used to estimate E-DII besides the one published by Shivappa et al. [44]; and only a few studies presented sex-specific E-DII/DII values. Despite these limitations, our E-DII range values in men are wider than the ones reported by the case-control study in Iran [73]. In women, our E-DII values are slightly lower than the ones reported by the case-control study in Germany; however, that study only included postmenopausal women [32]. No data on inflammatory biomarkers are available in the MCC-study; therefore, we could not conduct a validation analysis within our study. Finally, it should be acknowledged that several subgroups have been studied (i.e., E-DII and NEAC profile), and thus, some results might be due to chance.

## 5. Conclusions

In conclusion, the present study adds further evidence on the association between CRC risk and the inflammatory potential of the diet, as well as the combined effect of the inflammatory potential of the diet and its total dietary antioxidant capacity. The increased risk between these dietary scores and BC were not statistically significant. Additional studies with larger sample size should be performed to elucidate any possible association. 

## Figures and Tables

**Table 1:** Characteristics of colorectal cancer and breast cancer cases and controls in the Multi Case-Control Study in Spain (MCC-Spain). Numbers may differ due to missing values.

	Colorectal Cancer Study (*n* = 5299)			Breast Cancer Study (*n* = 3138)		
	Controls	CRC Cases	*p*-Value ^1^	Controls	BC Cases	*p*-Value ^1^
	mean(sd)/*N*(%)	mean(sd)/*N*(%)		mean(sd)/*N*(%)	mean(sd)/*N*(%)	
Sex						
Male	1781 (51.7%)	1183 (63.9%)	<0.001	-	-	
Female	1666 (48.3%)	669 (36.1%)		1652 (52.6)	1486 (47.4)	
E-DII score	−0.39 (1.9)	0.03 (1.9)	<0.001	−0.75 (1.8)	−0.50 (1.9)	<0.001
Total dietary NEAC (without coffee)						
TEAC (µmol TE/day)	4.01 (1.83)	3.96 (1.82)	0.33	3.74 (1.68)	3.75 (1.72)	0.98
Age (years)	63.2 (11.7)	66.8 (10.6)	<0.001^2^	59.0 (13.0)	56.2 (12.4)	<0.001
BMI (kg/m^2^)	26.6 (4.4)	27.6 (4.5)	<0.001	25.7 (4.8)	25.9 (4.6)	0.40
Energy intake (without alcohol; kcal/day)	1805 (535)	1900 (588)	<0.001	1727 (499)	1808 (529)	<0.001
Ethanol intake (g/day)	11.0 (15.9)	12.0 (19.3)	<0.001^2^	4.97 (8.2)	5.16 (9.3)	0.29 ^2^
Physical activity						
Inactive	1316 (38.6%)	949 (51.2%)	<0.001	628 (38.3%)	613 (41.3%)	0.40
Moderately active	501 (14.7%)	214 (11.6%)		305 (18.6%)	256 (17.2%)	
Active	416 (12.2%)	159 (8.59%)		214 (13.1%)	190 (12.8%)	
Very active	1178 (34.5%)	530 (28.6%)		491 (30.0%)	427 (28.7%)	
Education level						
Less than primary	604 (17.5%)	578 (31.2%)	<0.001	272 (16.5%)	209 (14.1%)	0.07
Primary	1120 (32.5%)	715 (38.6%)		500 (30.3%)	491 (33.0%)	
High school	992 (28.8%)	373 (20.1%)		520 (31.5%)	492 (33.1%)	
University	731 (21.2%)	186 (10.0%)		360 (21.8%)	294 (19.8%)	
Tobacco smoking						
Never smoker	1522 (44.3%)	766 (41.6%)	<0.001	991 (60.1%)	822 (55.5%)	0.004
Former smoker	1204 (35.1%)	747 (40.6%)		330 (20.0%)	294 (19.8%)	
Current smoker	709 (20.6%)	329 (17.9%)		329 (19.9%)	366 (24.7%)	
Family history ^3^						
Yes	297 (8.62%)	304 (16.4%)	<0.001	144 (8.72%)	218 (14.7%)	<0.001
No	2960 (85.9%)	1415 (76.4%)		1441 (87.2%)	1232 (82.9%)	
Missing-Unknown	190 (5.5%)	133 (7.2%)		67 (4.1%)	36 (2.4%)	
Use of NSAIDs/aspirin						
Yes	1334 (38.7%)	604 (32.6%)	<0.001	-	-	
No	2000 (58.0%)	1184 (63.9%)		-	-	
Missing	113 (3.3%)	64 (3.46%)				
Hormone replacement therapy use						
Never	-	-		1469 (88.9%)	1347 (90.6%)	0.15
Ever	-	-		126 (7.6%)	104 (7.0%)	
Not Known (or not remember)	-	-		57 (3.5%)	35 (2.4%)	
Oral contraceptive use						
No	-	-		839 (50.8%)	772 (52.0%)	0.52
Yes	-	-		812 (49.2%)	712 (48.0%)	
Age at menarche						
<13 years old	-	-		667 (40.4%)	630 (42.4%)	<0.001
≥13 years old	-	-		925 (56.0%)	837 (56.3%)	
Not known	-	-		60 (3.6%)	19 (1.3%)	
Number of children				1.9 (1.5)	1.7 (1.3)	<0.001 ^2^
Age at first pregnancy						
Nulliparous	-	-		310 (18.8%)	317 (21.5%)	0.33
<20 years	-	-		58 (3.53%)	59 (4.0%)	
20–24 years	-	-		407 (24.7%)	345 (23.4%)	
25–29 years	-	-		537 (32.6%)	454 (30.8%)	
>29 years	-	-		333 (20.2%)	301 (20.4%)	
Menopausal status						
Premenopausal	-	-		476 (28.8%)	532 (35.8%)	<0.001
Postmenopausal	-	-		1175 (71.2%)	953 (64.2%)	

BC, breast cancer; BMI, body mass index; CRC, colorectal cancer; E-DII; energy-adjusted dietary inflammatory index; MCC, Multi-case-control Spain study; Non-enzymatic antioxidant capacity, NEAC; NSADs, nonsteroidal anti-inflammatory drugs; TE, Trolox equivalents. ^1^ *p*-value obtained by Student t-test for continuous variables normally distributed or chi-squared test for categorical variables unless otherwise indicated. ^2^ *p*-value obtained by Wilcoxon rank-sum test for continuous variables non-normally distributed. ^3^ Only first relative degree colorectal cancers or breast cancer.

**Table 2:** Characteristics of participants in the control group (*n* = 3576) according to categories of the energy-adjusted dietary inflammatory index (E-DII) score (based on the quartile distribution in controls).

Variables		Q1 *N* = 894	Q2 *N* = 895	Q3 *N* = 892	Q4 *N* = 895	*p*-Value ^1^	*n*
	Men	(−5.11, −1.49)	(−1.49, −0.167)	(−0.167, 1.41)	(1.41, 5.47)		
	Women	(−5.64, −2.15)	(−2.15, −1.01)	(−1.01, 0.426)	(0.426, 5.12)		
		mean(sd) / *N*(%)	mean(sd) / *N*(%)	mean(sd) / *N*(%)	mean(sd) / *N*(%)		
Age		65.3 (10.8)	64.5 (11.1)	63.0 (12.0)	58.6 (13.0)	<0.001 ^2^	3576
BMI (kg/m^2^)		26.9 (4.4)	26.7 (4.43)	26.4 (4.3)	26.4 (4.52)	0.05	3576
Energy intake (without alcohol; kcal/day)		1627 (471)	1776 (495)	1841 (528)	1966 (581)	<0.001	3576
Ethanol intake (g/day)		9.4 (12.8)	9.5 (13.3)	11.3 (16.6)	13.6 (19.3)	<0.001 ^2^	3576
Total dietary NEAC (without coffee)							
TEAC (µmol TE/day)		4.76 (1.95)	4.25 (1.74)	3.82 (1.68)	3.17 (1.53)	<0.001	3450
Sex						1	3576
Men		455 (50.9%)	456 (50.9%)	455 (51.0%)	456 (50.9%)		
Women		439 (49.1%)	439 (49.1%)	437 (49.0%)	439 (49.1%)		
Education						<0.001	3576
Less than primary school		171 (19.1%)	169 (18.9%)	147 (16.5%)	130 (14.5%)		
Primary school		298 (33.3%)	299 (33.4%)	301 (33.7%)	245 (27.4%)		
Secondary school		241 (27.0%)	233 (26.0%)	259 (29.0%)	316 (35.3%)		
University		184 (20.6%)	194 (21.7%)	185 (20.7%)	204 (22.8%)		
Tobacco smoking						<0.001	3562
Never smoker		445 (49.9%)	427 (48.0%)	389 (43.7%)	328 (36.8%)		
Former smoker		316 (35.5%)	333 (37.5%)	312 (35.1%)	278 (31.2%)		
Current smoker		130 (14.6%)	129 (14.5%)	189 (21.2%)	286 (32.1%)		
Physical activity						<0.001	3540
Inactive		314 (35.4%)	303 (34.2%)	336 (38.1%)	405 (45.9%)		
Moderately active		119 (13.4%)	137 (15.4%)	135 (15.3%)	139 (15.7%)		
Active		107 (12.1%)	122 (13.8%)	102 (11.6%)	102 (11.6%)		
Very active		347 (39.1%)	325 (36.6%)	310 (35.1%)	237 (26.8%)		
Use of NSAIDs/aspirin						0.32	3576
Yes		346 (38.7%)	335 (37.4%)	353 (39.6%)	356 (39.8%)		
No		515 (57.6%)	522 (58.3%)	512 (57.4%)	519 (58.0%)		
Missing		33 (3.7%)	38 (4.3%)	27 (3.0%)	20 (2.2%)		
Hormone replacement therapy use						0.01	1754
Never		377 (85.9%)	383 (87.2%)	393 (89.9%)	407 (92.7%)		
Ever		38 (8.7%)	40 (9.1%)	28 (6.4%)	26 (5.9%)		
Not Known (or not remember)		24 (5.5%)	16 (3.6%)	16 (3.7%)	6 (1.4%)		
Oral contraceptive use						<0.001	1753
No		251 (57.2%)	247 (56.4%)	209 (47.8%)	196 (44.6%)		
Yes		188 (42.8%)	191 (43.6%)	228 (52.2%)	243 (55.4%)		
Age at menarche						0.30	1754
<13		172 (39.2%)	179 (40.8%)	166 (38.0%)	194 (44.2%)		
≥13		247 (56.3%)	240 (54.7%)	259 (59.3%)	231 (52.6%)		
Missing		20 (4.6%)	20 (4.6%)	12 (2.8%)	14 (3.2%)		
Age at first pregnancy						0.30	1742
Nulliparous		73 (16.7%)	83 (19.1%)	76 (17.6%)	91 (20.8%)		
<20 years		17 (3.9%)	11 (2.5%)	18 (4.2%)	15 (3.43%)		
20–24 years		104 (23.8%)	122 (28.0%)	104 (24.0%)	103 (23.6%)		
25–29 years		144 (33.0%)	142 (32.6%)	159 (36.7%)	133 (30.4%)		
>29 years		99 (22.7%)	77 (17.7%)	76 (17.6%)	95 (21.7%)		
Number of children		2.0 (1.5)	1.9 (1.4)	2.0 (1.5)	1.9 (1.4)	0.41 ^2^	1750
Menopausal status						<0.001	1753
Premenopausal		81 (18.5%)	100 (22.8%)	128 (29.4%)	184 (41.9%)		
Postmenopausal		358 (81.5%)	339 (77.2%)	308 (70.6%)	255 (58.1%)		

E-DII; energy-adjusted dietary inflammatory index; BMI, body mass index; Non-enzymatic antioxidant capacity, NEAC; NSADs, nonsteroidal anti-inflammatory drugs; TE, Trolox equivalents. ^1^ *p*-value obtained by Student *t*-test for continuous variables normally distributed or chi-squared test for categorical variables unless otherwise indicated. ^2^ *p*-value obtained by Wilcoxon rank-sum test for continuous variables non-normally distributed.

**Table 3:** Association between E-DII score and colorectal cancer in the MCC-Spain Study (*n* = 5299).

	Control/Cases	Models				E-DII Score Categories				*p* for Trend	*p* for Interaction
			Per 1-Point Increment in the E-DII Score			Q1	Q2	Q3	Q4		
					M	≤−1.49	(−1.49, −0.167)	(−0.167, 1.41)	>1.41		
					W	≤−2.15	(−2.15, −1.01)	(−1.01, 0.426)	>0.426		
			OR (95% CI)			OR (95% CI)	OR (95% CI)	OR (95% CI)	OR (95% CI)		
All	3447/1852	Simple	1.15 (1.11–1.19)			1.00 (ref)	1.59 (1.33–1.89)	1.99 (1.67–2.37)	2.03 (1.70–2.44)	<0.001	
	3399/1842	Final	1.14 (1.10–1.18)			1.00 (ref)	1.57 (1.31–1.89)	1.97 (1.64–2.35)	1.93 (1.60–2.32)	<0.001	
	3399/1596	Sensitivity	1.15 (1.11–1.19)			1.00 (ref)	1.66 (1.37–2.01)	2.06 (1.70–2.49)	2.02 (1.66–2.47)	<0.001	
CRC Subtype ^1^											
Colon cancer	3399/1122	Final	1.13 (1.08–1.17)			1.00 (ref)	1.53 (1.24–1.89)	1.86 (1.51–2.30)	1.81 (1.45–2.26)	<0.001	
Rectal cancer	3399/700		1.17 (1.11–1.22)			1.00 (ref)	1.69 (1.29–2.22)	2.23 (1.71–2.90)	2.27 (1.73–2.98)	<0.001	
Sex											
Men	1748/1174	Stratified 1	1.14 (1.09–1.19)			1.00 (ref)	1.72 (1.36–2.18)	2.02 (1.60–2.57)	2.14 (1.68–2.73)	<0.001	0.04
Women	1651/668		1.12 (1.05–1.18)			1.00 (ref)	1.39 (1.05–1.86)	1.95 (1.47–2.59)	1.57 (1.16–2.13)	<0.001	
BMI (kg/m^2^)											
Normal weight	1308/544	Stratified 2	1.12 (1.06–1.19)			1.00 (ref)	1.52 (1.09–2.12)	1.75 (1.26–2.44)	1.95 (1.39–2.73)	<0.001	0.87
Overweight and Obese	2091/1298		1.14 (1.10–1.19)			1.00 (ref)	1.58 (1.27–1.97)	2.06 (1.66–2.55)	1.90 (1.51–2.37)	<0.001	
Physical activity ^2^											
Inactive	1310/945	Stratified 3	1.17 (1.08–1.20)			1.00 (ref)	1.69 (1.28–2.24)	2.31 (1.76–3.03)	2.05 (1.55–2.70)	<0.001	0.52
Active	2089/897		1.15 (1.09–1.20)			1.00 (ref)	1.50 (1.18–1.91)	1.78 (1.39–2.26)	1.92 (1.49–2.47)	<0.001	
Use of NSAIDs/aspirin											
Yes	1298/601	Stratified 4	1.14 (1.07–1.20)			1.00 (ref)	1.73 (1.26–2.37)	1.94 (1.42–2.65)	1.97 (1.43–2.72)	<0.001	0.25
No	1990/1178		1.13 (1.08–1.18)			1.00 (ref)	1.49 (1.18–1.88)	1.97 (1.57–2.47)	1.83 (1.44–2.32)	<0.001	
Tobacco smoking											
Current/Former smokers	1893/1076	Stratified 5	1.09 (1.05–1.14)			1.00 (ref)	1.61 (1.26–2.07)	1.87 (1.47–2.39)	1.72 (1.34–2.20)	<0.001	0.58
Never smokers	1506/766		1.18 (1.12–1.25)			1.00 (ref)	1.53 (1.17–2.00)	2.05 (1.57–2.68)	2.11 (1.58–2.82)	<0.001	

BMI, body mass index; CRC, colorectal cancer; E-DII; energy-adjusted dietary inflammatory index; M, men; MCC, Multi-case-control Spain study; NEAC, dietary non-enzymatic total antioxidant capacity; NSADs, nonsteroidal anti-inflammatory drugs; W, women. Simple model: Logistic regression analyses adjusted for sex (except in models stratified by gender), age, educational level, and study area. Final model: Logistic regression analyses adjusted for sex, age, educational level, study area, family history of colorectal cancer, tobacco smoking, physical activity, BMI, and NSAIDs/aspirin use. Sensitivity analysis excluding cases that had more than 6 months between the data of diagnosis and the interview: Logistic regression analyses adjusted for the same variables as Final model. Stratified 1: Logistic regression analyses adjusted, age, educational level, study area, family history of colorectal cancer, tobacco smoking, physical activity, BMI, and NSAIDs/aspirin use. Stratified 2: Logistic regression analyses adjusted for sex, age, educational level, study area, family history of colorectal cancer, tobacco smoking, physical activity, and NSAIDs/aspirin use. Stratified 3: Logistic regression analyses adjusted for sex, age, educational level, study area, family history of colorectal cancer, tobacco smoking, BMI, and NSAIDs/aspirin use. Stratified 4: Logistic regression analyses adjusted for sex, age, educational level, study area, family history of colorectal cancer, tobacco smoking, physical activity, and BMI. Stratified 5: Logistic regression analyses adjusted for sex, age, educational level, study area, family history of colorectal cancer, physical activity, BMI, and NSAIDs/aspirin use. ^1^ In 20 colorectal cancer cases, tumor subtype was not available, hence were excluded. ^2^ Categorized as inactive and active (including "moderately active", "active”, and "very active").

**Table 4:** Association between E-DII score and breast cancer in the MCC-Spain Study (*n* = 3138) .

	Control/Case	Models		E-DII Score Categories				*p* for Trend	*p* for Interaction
			Per 1-Point Increment in the E-DII Score	Q1	Q2	Q3	Q4		
				≤−2.15	(−2.15, −1.01)	(−1.01, 0.426)	>0.426		
			OR (95% CI)	OR (95% CI)	OR (95% CI)	OR (95% CI)	OR (95% CI)		
All women	1652/1486	Simple	1.05 (1.01–1.09)	1.00 (ref)	1.17 (0.95–1.45)	1.13 (0.92–1.39)	1.25 (1.02–1.55)	0.06	
	1628/1471	Final	1.04 (1.00–1.08)	1.00 (ref)	1.16 (0.94–1.43)	1.13 (0.91–1.39)	1.22 (0.99–1.52)	0.10	
	1628/1140	Sensitivity	1.03 (0.99–1.08)	1.00 (ref)	1.17 (0.93–1.47)	1.09 (0.87–1.37)	1.19 (0.94–1.50)	0.24	
BC subtypes									
HR+ ^1^	1628/986	Final	1.04 (0.99–1.09)	1.00 (ref)	1.17 (0.92–1.49)	1.17 (0.93–1.49)	1.22 (0.95–1.55)	0.14	
HER2+ ^1^	1628/251	Final	1.04 (0.96–1.13)	1.00 (ref)	1.85 (1.21–2.83)	1.27 (0.81–1.98)	1.56 (1.01–2.04)	0.24	
TN^1^	1628/105	Final	1.02 (0.91–1.14)	1.00 (ref)	0.67 (0.37–1.23)	0.78 (0.44–1.39)	0.99 (0.56–1.75)	0.97	
Menopausal status									
Premenopausal	469/526	Stratified 1	1.01 (0.94–1.08)	1.00 (ref)	0.94 (0.60–1.47)	0.97 (0.64–1.48)	1.05 (0.70–1.57)	0.71	0.33
Postmenopausal	1159/945		1.06 (1.01–1.12)	1.00 (ref)	1.23 (0.97–1.58)	1.19 (0.93–1.54)	1.30 (0.99–1.69)	0.08	
HRT use									
Never	1448/1335	Stratified 2	1.04 (1.00–1.08)	1.00 (ref)	1.16 (0.93–1.46)	1.16 (0.92–1.45)	1.22 (0.97–1.53)	0.11	0.54
Ever	125/103		1.12 (0.93–1.34)	1.00 (ref)	0.88 (0.38–2.05)	1.12 (0.47–2.64)	1.47 (0.62–3.49)	0.35	
OC use									
No	828/764	Stratified 3	1.05 (0.99–1.11)	1.00 (ref)	1.25 (0.94–1.66)	1.35 (1.01–1.81)	1.20 (0.88–1.63)	0.18	0.87
Yes	800/707		1.04 (0.98–1.10)	1.00 (ref)	1.10 (0.79–1.52)	0.97 (0.71–1.33)	1.25 (0.91–1.71)	0.25	
BMI (kg/m^2^)									
Normal weight	843/718	Stratified 4	1.04 (0.98–1.10)	1.00 (ref)	0.88 (0.64–1.21)	1.01 (0.74–1.38)	1.20 (0.88–1.63)	0.15	0.99
Overweight and Obese	785/753		1.05 (0.99–1.12)	1.00 (ref)	1.49 (1.11–2.01)	1.25 (0.93–1.68)	1.29 (0.95–1.76)	0.23	
Physical activity ^2^									
Inactive	625/608	Stratified 5	1.06 (0.99–1.12)	1.00 (ref)	1.92 (1.33–2.78)	1.63 (1.14–2.34)	1.48 (1.04–2.10)	0.13	0.92
Active	1003/863		1.03 (0.97–1.09)	1.00 (ref)	0.93 (0.71–1.21)	0.90 (0.69–1.18)	1.14 (0.86–1.51)	0.45	
Tobacco smoking									
Current/Former smokers	647/656	Stratified 6	1.05 (0.98–1.11)	1.00 (ref)	0.85 (0.59–1.22)	1.01 (0.71–1.44)	1.15 (0.81–1.61)	0.21	0.15
Never smokers	981/815		1.03 (0.97–1.09)	1.00 (ref)	1.40 (1.07–1.82)	1.19 (0.91–1.57)	1.19 (0.89–1.59)	0.39	

BMI, body mass index; CRC, colorectal cancer; E-DII; energy-adjusted dietary inflammatory index; HRT, hormone replacement therapy; MCC, Multi-case-control Spain study; NSADs, nonsteroidal anti-inflammatory drugs; OC, oral contraceptive. Simple model: Logistic regression analyses adjusted for age, study area, and educational level. Final model: Logistic regression analyses adjusted for age, study area, educational level, family history of breast cancer, tobacco smoking, HRT use, OC use, age at menarche, age at first pregnancy, number of children, menopausal status, physical activity, and BMI. Sensitivity analysis excluding cases that had more than six months between the data of diagnosis and the interview: Logistic regression analyses adjusted for the same variables as Final model. Stratified 1: Logistic regression analyses adjusted for age, study area, educational level, family history of breast cancer, tobacco smoking, HRT use, OC use, age at menarche, age at first pregnancy, number of children, physical activity, and BMI. Stratified 2: Logistic regression analyses adjusted for age, study area, educational level, family history of breast cancer, tobacco smoking, OC use, age at menarche, age at first pregnancy, number of children, menopausal status, physical activity, and BMI. Stratified 3: Logistic regression analyses adjusted for age, study area, educational level, family history of breast cancer, tobacco smoking, HRT use, age at menarche, age at first pregnancy, number of children, menopausal status, physical activity, and BMI. Stratified 4: Logistic regression analyses adjusted for age, study area, educational level, family history of breast cancer, tobacco smoking, HRT use, OC use, age at menarche, age at first pregnancy, number of children, menopausal status, and physical activity. Stratified 5: Logistic regression analyses adjusted for age, study area, educational level, family history of breast cancer, tobacco smoking, HRT use, OC use, age at menarche, age at first pregnancy, number of children, menopausal status, and BMI. Stratified 6: Logistic regression analyses adjusted for age, study area, educational level, family history of breast cancer, HRT use, OC use, age at menarche, age at first pregnancy, number of children, menopausal status, physical activity, and BMI. ^1^ HR+: hormone receptor positive tumors (ER+ or PR+ with HER2-); HER2+: human epidermal growth factor receptor positive tumors, independent of ER or PR; TN: triple negative tumors (ER-, PR-, and HER2-). In 131 breast cancer cases, tumor subtype was not available, hence were excluded. ^2^ Categorized as inactive and active (including "moderately active", "active”, and "very active")

**Table 5:** Association between E-DII score and dietary non-enzymatic antioxidant capacity (NEAC) and colorectal and breast cancer in the MCC-Spain Study.

	**Control/Case**	**Models**		**E-DII+NEAC Score Categories**					***p*** **for Trend**
					**≤2**	**3**	**4**	**≥5**	
			**Per 1-Point Increment in the E-DII+NEAC Score**	**M: mean E-DII**	−1.49	−0.12	0.80	2.15	
				**W: mean E-DII**	−2.12	−0.89	−0.16	1.28	
				**M: mean TEAC**	5.68	4.11	3.45	2.39	
				**W: mean TEAC**	4.93	3.68	3.02	2.19	
			**OR (95% CI)**		**OR (95% CI)**	**OR (95% CI)**	**OR (95% CI)**	**OR (95% CI)**	
CRC	3312/1798	Final-CRC	1.10 (1.06–1.14)		1.00 (ref)	1.31 (1.10–1.55)	1.32 (1.10–1.58)	1.48 (1.26–1.74)	<0.001
CRC Subtype ^1^									
Colon cancer	3312/1100	Final-CRC	1.09 (1.05–1.14)1.		1.00 (ref)	1.31 (1.07–1.60)	1.29 (1.04–1.60)	1.46 (1.21–1.76)	<0.001
Rectal cancer	3312/678	Final-CRC	1.11 (1.06–1.17)		1.00 (ref)	1.29 (1.01–1.65)	1.34 (1.04–1.73)	1.49 (1.19–1.87)	<0.001
BC	1585/1418	Final-BC	1.02 (0.98–1.07)		1.00 (ref)	0.98 (0.80–1.22)	1.03 (0.82–1.28)	1.09 (0.90–1.32)	0.39
Menopausal status									
Premenopausal	464/506	Stratified 1	0.98 (0.91–1.06)		1.00 (ref)	1.04 (0.68–1.57)	0.88 (0.58–1.33)	0.91 (0.66–1.27)	0.50
Postmenopausal	1121/912	Stratified 1	1.05 (0.99–1.11)		1.00 (ref)	0.95 (0.74–1.22)	1.12 (0.85–1.48)	1.22 (0.95–1.56)	0.10

BC, breast cancer; CRC, colorectal cancer; E-DII; energy-adjusted dietary inflammatory index; M, men; MCC, Multi-case-control Spain study; NEAC, dietary non-enzymatic total antioxidant capacity; TE, Trolox equivalents; W, women, Final model (CRC): Logistic regression analyses adjusted for sex, age, educational level, study area, family history of colorectal cancer, tobacco smoking, physical activity, BMI, and NSAIDs/aspirin use. Final model (BC): Logistic regression analyses adjusted for age, study area, educational level, family history of breast cancer, tobacco smoking, HRT use, OC use, age at menarche, age at first pregnancy, number of children, menopausal status, physical activity, and BMI. Stratified 1: Logistic regression analyses adjusted for age, study area, educational level, family history of breast cancer, tobacco smoking, HRT use, OC use, age at menarche, age at first pregnancy, number of children, physical activity, and BMI. ^1^ In 20 colorectal cancer cases, tumor subtype was not available, hence were excluded.

## Supplementary Materials

The following are available online at https://www.mdpi.com/2072-6643/11/6/1406/s1, Table S1: Association E-DII and dietary NEAC and colorectal and breast cancer in the MCC-Spain Study. Table S2: Stratified analyses: OR and 95%CI for E-DII score and dietary NEAC and colorectal and breast cancer risk in the MCC-Spain Study
